# RhoA-GTPase Modulates Neurite Outgrowth by Regulating the Expression of Spastin and p60-Katanin

**DOI:** 10.3390/cells9010230

**Published:** 2020-01-16

**Authors:** Dandan Tan, Haowen Zhang, Junyao Deng, Jingmin Liu, Jinkun Wen, Lixia Li, Xianghai Wang, Mengjie Pan, Xiaofang Hu, Jiasong Guo

**Affiliations:** 1 Guangdong Provincial Key Laboratory of Construction and Detection in Tissue Engineering, Southern Medical University, Guangzhou 510515, China; dandantan@yeah.net (D.T.); haowenzhang218710@outlook.com (H.Z.); gary.d@163.com (J.D.); liujingmin1986@163.com (J.L.); wenjinkun@yeah.net (J.W.); lixialilpf@yeah.net (L.L.); wangxianghai@i.smu.edu.cn (X.W.); mzp247@psu.edu (M.P.); xiaofang4231@163.com (X.H.); 2 Department of Histology and Embryology, Southern Medical University, Guangzhou 510515, China; 3 Department of Human Anatomy, Guangdong Medical University, Zhanjiang 524023, China; 4 Guangzhou Regenerative Medicine and Health Guangdong Laboratory, Guangzhou 510515, China; 5 Key Laboratory of Mental Health of the Ministry of Education, Guangdong-Hong Kong-Macao Greater Bay Area Center for Brain Science and Brain-Inspired Intelligence, Guangdong Province Key Laboratory of Psychiatric Disorders, Guangzhou 510515, China

**Keywords:** neurite outgrowth, RhoA signaling pathway, spastin, p60-katanin, microtubule-severing proteins, Glu-tubulin

## Abstract

RhoA-GTPase (RhoA) is widely regarded as a key molecular switch to inhibit neurite outgrowth by rigidifying the actin cytoskeleton. However, during neurite outgrowth, whether and how microtubule dynamics are regulated by RhoA remains to be elucidated. Herein, CT04 and Y27632 were used to inactivate RhoA and its downstream effector Rho-associated coiled coil-forming kinase (ROCK), while the RhoAQ63L lentiviral vector was utilized to overexpress the constitutively activated RhoA in dorsal root ganglion (DRG) neurons or neuronal differentiated PC12 cells. The current data illustrate that the RhoA signaling pathway negatively modulates neurite outgrowth and elevates the expression of Glu-tubulin (a marker for a stabilized microtubule). Meanwhile, the microtubule-severing proteins spastin and p60-katanin were downregulated by the RhoA signaling pathway. When spastin and p60-katanin were knocked down, the effects of RhoA inhibition on neurite outgrowth were significantly reversed. Taken together, this study demonstrates that the RhoA pathway-mediated inhibition of neurite outgrowth is not only related to the modulation of microfilament dynamics but is also attributable to the regulation of the expression of spastin and p60-katanin and thus influences microtubule dynamics.

## 1. Introduction

Neurite outgrowth is a requisite process for the development and regeneration of the nervous system, through which connections between neurons and their targets can be built up [1,2]. A wealth of evidence has demonstrated that cytoskeletal (actin microfilament and microtubules) dynamics play central roles in neurite outgrowth [1,3]. Many studies have suggested that the assembly of actin filaments in the growth cone is essential for axon guidance [4,5]. On the other hand, microtubules are also important for growth cone guidance because their polarized invasion into the peripheral domain on one side of the growth cone is necessary to make the growth cone turn in that direction [6,7,8]. Moreover, microtubules are crucial for axon elongation, organelle transport, and growth cone motility related to their dynamic instability [9,10]. The mechanisms of actin filaments dependent on growth cone steering and axonal regeneration, via signaling cascades, are well documented [4,5]. Meanwhile, the underlying molecular mechanism of neurite outgrowth through extracellular signals is far from fully understood.

In the micro-environment of growing neurites, especially in the injured nerve system, there are many kinds of extracellular inhibitory molecules, such as chondroitin sulfate proteoglycans (CSPGs), myelin-associated glycoprotein (MAG), Lingo-1, Nogo, oligodendrocyte-myelin glycoprotein (OMgp), and so on [11,12,13]. Existing evidence has suggested that the small GTPase RhoA subfamily (RhoA) is an intracellular convergence point for most extracellular inhibitory signals [14,15,16]. Previous studies have reported that the mechanism of RhoA action as a pivotal inhibitory signaling pathway in developing and regenerating neurons can be attributed to actin microfilament rearrangements, since RhoA facilitates the formation of actin stress fibers and focal adhesions through its downstream effector, Rho-associated coiled coil-forming kinase (ROCK) [15,17]. For the above reasons, the RhoA/ROCK pathway has been identified as a promising therapeutic target to promote nerve regeneration [17,18,19,20].

Recently, several studies have suggested that the RhoA signaling pathway is also involved in the regulation of microtubule dynamics in many cells [21,22], including neurons. For example, Guo’s group reported that Y27632, a specific inhibitor of ROCK, can change the microtubule distribution in cultured hippocampal neurons [23]. Rozés Salvador et al. demonstrated that the axon growth inhibition induced by the mAb-targeting ganglioside GD1a/GT1b involved the negative regulation of the microtubule cytoskeleton via a RhoA/ROCK-dependent pathway [24]. However, to date, our understanding of how the RhoA signaling pathway regulates microtubule dynamics during neurite outgrowth remains unclear. To address this issue, we used CT04 and Y27632 to inhibit the activation of RhoA and ROCK, respectively, and utilized a lentiviral vector to overexpress the constitutively activated RhoA in dorsal root ganglion (DRG) neurons and neuronally differentiated PC12 cells. The findings of the present study are the first to demonstrate that the RhoA signaling pathway can modulate the expression of the microtubule-severing proteins spastin and p60-katanin, which may contribute to the regulation of microtubule dynamics and thus influence neurite outgrowth. The present results enrich our understanding of the mechanism of the RhoA signaling pathway on neurite outgrowth during neuronal development and regeneration.

## 2. Materials and Methods

### 2.1. Cell Culture

#### 2.1.1. Culture of Primary DRG Neurons

The procedures in this experimental study were carried out in accordance with National Institutes of Health (NIH) guidelines for the care and use of laboratory animals (NIH Publications) and approved by the Animal Experimental Ethics Committee of the Southern Medical University (SMU-L2015081, proved on 2015.10.15), Guangdong Province, China. All efforts were made to minimize animal suffering and usage. Rat DRG neurons were isolated and cultured based on our previous report [25]. Briefly, DRGs from all spinal levels were aseptically isolated from 1–3 days postnatal Sprague Dawley (SD) rats (provided by the Experimental Animal Center of Southern Medical University). The epineurium of each ganglion was stripped off in cold Hank’s balanced salt solution (HBSS, Gibco , Grand Island, NY, USA) under a stereomicroscope (Olympus, Tokyo, Japan). DRGs were dissociated by 0.125% Trypsin-EDTA (Gibco, Grand Island, NY, USA) digestion at 37 ℃ for 30 min with efficient pipetting. Following digestion and dissociation, the cells were centrifuged for 10 min at 106× *g* and resuspended in DMEM/F12 (Corning, New York, NY, USA) containing 1% fetal bovine serum (FBS, Corning, New York, NY, USA) and 100 mg/mL penicillin–streptomycin (Gibco, Grand Island, NY, USA), plated at a density of 1 × 10^4^ cells/mL on 0.1 mg/mL poly-L-lysine (PLL, Sigma, St. Louis, MO, USA) precoated glass coverslips (Fisher Scientific, Pittsburgh, PA, USA). The cells were incubated at 37 °C and 5% CO_2_ for further studies.

#### 2.1.2. Culture and Neuronal Induction of PC12 Cells

The PC12 cells used in this study were a gift from Prof. Zhou L (GHM Institute of CNS Regeneration, Jinan University, Guangzhou, China). The cells were maintained at 37 °C in a 5% CO_2_ humidified atmosphere in DMEM/F12 supplemented with 10% FBS and 100 mg/mL penicillin–streptomycin (Gibco, Grand Island, NY, USA). For immunofluorescence studies, the cells were planted at a density of 1 × 10^4^ cells/mL on coverslips and cultured overnight in the above medium. For the quantitative real-time PCR (RT-PCR) and Western blot studies, the cells were planted at a density of 1 × 10^5^ cells/mL in 60 mm dishes. The next day, the medium was replaced with DMEM/F12 containing 1% FBS. After 24 h, the medium was replaced with a neuronal inductive medium (DMEM/F12 containing 1% FBS, 50 ng/mL nerve growth factor (NGF, 2.5S, Millipore, Burlington, MA, USA), 20 ng/mL brain-derived neurotrophic factor (BDNF, Gibco, Grand Island, NY, USA), and 15 μM Forskolin (Sigma, St. Louis, MO, USA) to induce neurogenic differentiation. The medium was refreshed every 2 days. Six days later, the cells were collected for further studies. 

### 2.2. Pharmacological Treatment

To investigate the effects of the inhibition of the RhoA signaling pathway, the DRG neurons and neuronal differentiated PC12 cells were treated with 2 μg/mL CT04 (RhoA inhibitor, Cytoskeleton, Denver, CO, USA) or 50 μM Y27632 (ROCK inhibitor, Selleck, Houston, TX, USA) for 24 h. In the designed experiments, 10 μM MK2206 (a specific inhibitor of AKT, Selleck, Houston, TX, USA) or 10 μM SC79 (a specific activator of AKT, Selleck, Houston, TX, USA) were added into the culture medium and maintained for 24 h.

### 2.3. Cell Transfection and Lentivirus Infection

Lentiviruses (LV) and shRNAs were constructed by Obio Technology (Shanghai, China). 5′-GGCTAAGGACCGTTTACAAA-3′ and 5′-GGTCTATTATCAGGGAGTT-3′ were selected to target the mRNA of spastin and p60-katanin, respectively. 5′-TTCTCCGAACGTGTCACGT-3′ was used as the controlled sequence. The spastin or p60-katanin shRNA-expression cassette was digested with the enzymes Age I and EcoR I and then cloned into the same sites in the lentiviral vector pLKD-CMV-eGFP-U6-shRNA. A lentivirus expressing constitutively activated mutants of RhoA (pLenti-Ubc-EGFP-P2A-3FLAG-RhoA-Q63L) was also serviced by Obio Technology.

For the lentivirus infection of the DRG neurons, 1 × 10^4^ cells/mL were exposed to LV-constitutively activated RhoA (RhoAQ63L) or an empty lentiviral vector (LV-control) at a final concentration of 1 × 10^6^ TU/mL for 24 h. The culture medium was then replaced with DMEM/F12 containing 1% FBS for 3 days before further assessments. For the lentivirus infection of PC12 cells, cells were plated with 1 × 10^5^ cells/mL in 60 mm culture dishes in DMEM/F12 with 10% FBS for 24 h. Then, the cells were induced as previously described, followed by infection with RhoAQ63L, spastin-shRNA, p60-katanin-shRNA, or the LV-control at a final concentration of 8 × 10^6^ TU/mL for 24 h to allow the expression of the transgene. After discarding the lentiviruses, the cells were allowed to grow for 3 days before further assessments. The effectively transfected cells were identified by their expression of GFP. 

### 2.4. Immunocytochemistry

At the designated time points, the subjected cells were fixed with 4% (*w/v*) paraformaldehyde (PFA) for 20 min and washed three times in 0.01 M PBS. This was followed by permeabilization with 0.5% Triton X-100 (Sigma, St. Louis, MO, USA) for 30 min. Cells were then blocked with 5% bovine serum albumin (BSA, GBCBIO Technologies, Guangzhou, China) in 0.01 M PBS for 1 h, followed by incubation with primary antibodies diluted in 1% BSA overnight at 4 °C. Alexa 488 and/or 568 fluorescent-conjugated secondary antibodies (1:400, Life Technologies, Gaithersburg, MD, USA) were applied for 2 h at room temperature, and the nuclei of all cells were counterstained by 1 μg/mL 4′,6-diamidino-2-phenylindole (DAPI, Sigma, St. Louis, MO, USA) for 2 min. The primary antibodies used for immunocytochemistry were as follows: mouse anti-β-tubulin III (1:400, Sigma, St. Louis, MO, USA), mouse anti-spastin (1:200, Sigma, St. Louis, MO, USA), and rabbit anti-p60-katanin (1:200, Proteintech, Chicago, IL, USA). After DAPI counterstaining, the cells were mounted with an anti-fading mounting medium (Vector, Burlingame, CA, USA), and images were captured by a fluorescent microscope (Leica, Wetzlar, Germany). Five images with high-power fields at 400x magnification per well were analyzed, and all images used for the quantification of fluorescence intensities were acquired and generated using identical imaging parameters (e.g., laser power, exposure, pixel saturation, gain, etc.). The fluorescence intensities of spastin and p60-katanin in each cell were assessed using the Image-Pro Plus 6.0 software (Media Cybernetics, Rockville, MD, USA). Finally, the obtained single-channel fluorescent images were merged using the Photoshop 6.0 software (Adobe, San Jose, CA, USA).

### 2.5. Measurement of Neurite Outgrowth

DRG neurons and neuronal differentiated PC12 cells were visualized by anti-β-tubulin-III immunostaining under a fluorescence microscope. The cells with neurites longer than the diameter of the cell body were measured as positive neurite-bearing cells, as previously described [26]. The average number and length of the neurites in each cell were assessed for all neurite-bearing cells in each field. Neurite outgrowth was measured as the average of 100 cells from each group per experiment, and each experiment was conducted in triplicate.

### 2.6. Western Blotting

For Western blotting, subjected cells were washed twice with ice-cold PBS, scraped, and lysed in a RIPA buffer (GBCBIO Technologies, Guangzhou, China) containing a protease inhibitor cocktail (1:100, Cell Signaling Technology, Danvers, MA, USA ). Lysates were incubated on ice for 30 min and centrifuged (14,462 × *g*, 20 min, 4 °C) in order to collect the supernatant. Extracts were separated in an SDS-PAGE sample buffer on 10% SDS-PAGE gels and transferred to a polyvinylidene difluoride (PVDF) membrane (Bio-Rad, Hercules, CA, USA). Blots were blocked with 5% BSA for 1 h and incubated with primary antibodies overnight at 4 °C and then incubated with HRP-conjugated secondary antibodies (Promega, Madison, WI, USA) for 2 h at room temperature. The following primary antibodies were used: rabbit anti-GAPDH (1:3000, Multi Sciences, Hangzhou, China), mouse anti-β-actin (1:2000, Multi Sciences, Hangzhou, China), mouse anti-α-tubulin (1:2000, Abcam, Cambridge, UK), rabbit anti-detyrosinated alpha tubulin (Glu-tubulin, 1:500, Abcam, Cambridge, UK), mouse anti-spastin (1:1000, Sigma, St. Louis, MO, USA), rabbit anti-p60-katanin (1:500, Proteintech, Chicago, IL, USA), rabbit anti-phospho-p38 (1:1000, Cell Signaling Technology, Danvers, MA, USA), rabbit anti-p38 (1:1000, Cell Signaling Technology, Danvers, MA, USA), rabbit anti-PTEN (1:1000, Cell Signaling Technology, Danvers, MA, USA), rabbit anti-PI3K (1:1000, Cell Signaling Technology, Danvers, MA, USA), rabbit anti-phospho-AKT (Ser473) (1:1000, Cell Signaling Technology, Danvers, MA, USA), and rabbit anti-AKT (1:1000, Cell Signaling Technology, Danvers, MA, USA). The Western blotting for each protein was repeated three times for each test. Immunoreactive protein bands were detected and imaged by enhanced chemiluminescence (ECL, Millipore, Burlington, MA, USA) using a Lumazone system (Roper, Trenton, NJ, USA). The integrated optical density (IOD) of each lane was quantified with the Image-Pro Plus software, and the expression levels of the targeted proteins were calculated by dividing the IOD of the targeted proteins by the IOD of the GAPDH or β-actin.

### 2.7. RNA Extraction and Quantitative Real-Time PCR

RNA extraction was performed using a Trizol reagent (Tiangen, Beijing, China) following the manufacturer’s protocol. The transcription of RNA to cDNA was carried out with the iScriptTM cDNA Synthesis Kit (Bio-rad, Hercules, CA, USA), according to the manufacturer’s instructions, for 60 min at 42 °C and for 10 min at 99 °C, resulting in 25 μL of cDNA. The quantitative real-time PCR (RT-PCR) of the cDNA from the neuronally differentiated PC12 cells was performed on a Bio-rad iCycler at a final volume of 10 μL with 5 μL SYBR Green (Tiangen, Beijing, China), 1.7 μL sterile water, 0.8 μL of each primer (2.5 μM), and a 2.5 μL cDNA template (a 3 min denaturing step followed by 40 cycles of 30 s at 95 °C and 60 s at 60 °C). The primer sequences used in this report are listed as follows: *GAPDH* as a housekeeping gene control: forward, 5′-TGCACCACCAACTGCTTAG-3′; reverse, 5′-GGATGCAGGGATGATGTT-3′; *SPAST*: forward, 5′-GAGACAGTGGATGGTAGAAG-3′; reverse, 5′-GTGCTAATGGAACAGTAGGA-3′; *KATNA1*: forward, 5′-CAGGAAGCAGTGGTGTTA-3′; reverse, 5′-GGATGAAGAGACATTGAAGAAC-3′; *KATNB1*: forward, 5′-GAATGCGGAGGACTACAAT-3′; reverse, 5′-TGCTGTGGCTATGTCATC-3′; *FIGN*: forward, 5′-CCTCGCTCATCCAATACAA-3′; reverse, 5′-CTCCTTAATAACCGCCTTCA-3′.

### 2.8. Statistical Analysis

All values are presented as the mean ± standard error of the mean (SEM), and SPSS 20.0 software (IBM, Armonk, NY, USA) was used for the statistical analysis. A one-way ANOVA (Bonferroni post hoc comparison) was used to determine the statistical significance of the differences among multiple groups, and a *p*-value less than 0.05 was considered statistically significant.

## 3. Results

### 3.1. The RhoA Signaling Pathway Negatively Modulates Neurite Outgrowth in DRG Neurons

**Figure 1:**
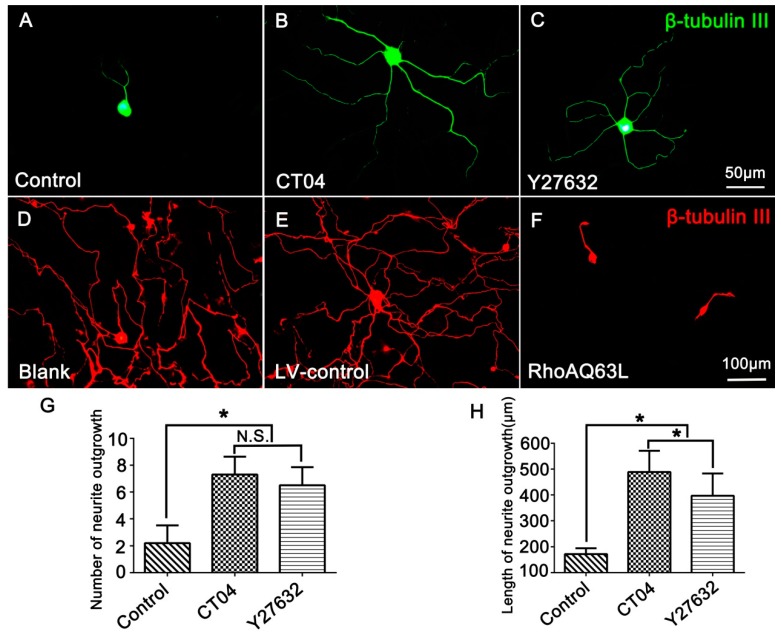
The RhoA signaling pathway negatively modulates neurite outgrowth in DRG neurons. (**A**–**C**) Representative images of β-tubulin III-positive neurons (green) in different drug treatment groups. Quantitative analyses of the number (**G**) and length (**H**) of the neurites of each neuron in different groups. Compared with the control group, CT04 and Y27632 treatments remarkably increased the number and length of the neurites. (**D**–**F**) Representative immunofluorescence staining for β-tubulin III (red) showed that compared with the Blank and LV-control groups, the overexpression of constitutively activated RhoA (RhoAQ63L transfection) dramatically reduced neurite outgrowth in the DRG neurons. Data are presented as the mean ± standard error of the mean (SEM) (* indicates *p* < 0.05, meaning a statistically significant difference, while N.S. indicates nonsignificance).

In order to investigate the effect of RhoA on neurite outgrowth, the cultured DRG neurons were treated with CT04 (a widely used RhoA inhibitor) for 24 h. The results showed that CT04 treatment of DRG neurons exerts a statistically significant, positive neurite outgrowth effect. The mean number of neurites per cell was elevated more than threefold (Figure 1A,B,G), and the length of the neurite increased more than fivefold (Figure 1A,B,H). Furthermore, to further confirm whether ROCK was involved in the regulation of neurite outgrowth, the DRG neurons were treated with Y27632. Likewise, the inhibition of ROCK by Y27632 produced a similar effect on the DRG neurons (Figure 1A,C). The average number of neurites per cell and the neurite length were elevated nearly threefold (Figure 1G) and more than fourfold (Figure 1H), respectively. To further specify the role of the RhoA/ROCK pathway in neurite outgrowth, we examined whether overexpression of constitutively activated RhoA inhibited neurite outgrowth. As expected, overexpression of constitutively activated RhoA by transfection with LV-RhoAQ63L, resulting in dramatic neurite retraction (Figure 1D–F). Notably, Figure 1A shows a representative image of cells from the Control group without drug treatment to compare the effects of the CT04 and Y27632 treatments, while Figure 1D shows a representative image of the cells from the Blank control group without lentivirus transfection, which was set to analyze the efficiency of the LV-control and LV-RhoAQ63L transfection. Due to the different schedules of drug treatment and lentiviral vector treatment, the DRG neurons in the Blank group were cultured for 3 days longer than those in the Control group to allow expression of the transgene. Consequently, the neurites in the Blank group were dramatically longer than those in the Control group. Moreover, the interweaving of neurites makes it impossible to calculate the length and number of the neurites of each neuron. Therefore, only the number and length of the neurites in the Control, CT04, and Y27632 groups were quantified.

### 3.2. The RhoA Pathway Negatively Regulates Neurite Outgrowth in Neuronally Differentiated PC12 Cells

**Figure 2:**
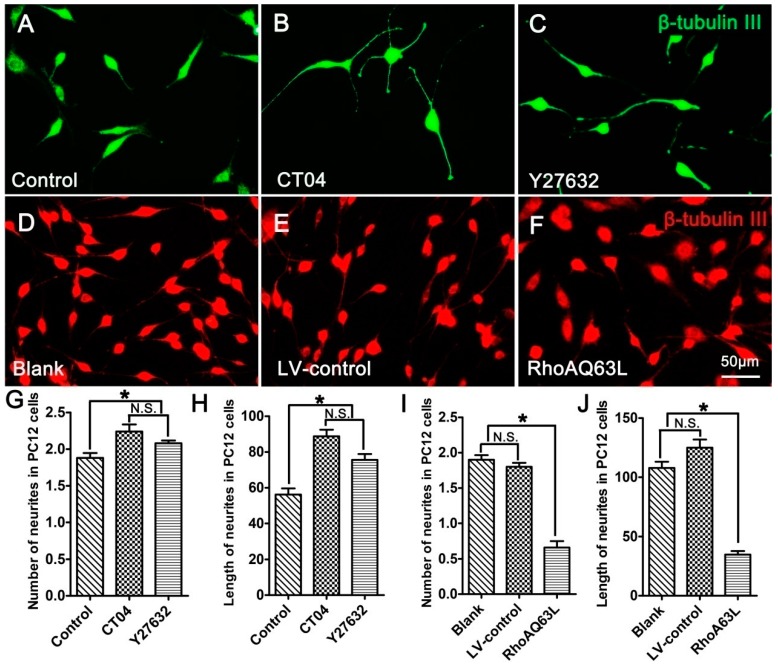
The RhoA signaling pathway negatively regulates neurite outgrowth in neuronally differentiated PC12 cells. (**A**–**C**) Representative immunofluorescence images of β-tubulin III-positive differentiated PC12 cells in different drug treatment groups. Quantitative analyses of the average number (**G**) and length (**H**) of the neurites of each cell in different groups. Compared with the control group, CT04 or Y27632 treatment increased the number and length of the neurites. (**D**–**F**,**I**,**J**) Representative immunofluorescence staining for β-tubulin III (red) revealed that overexpression of the constitutively activated RhoA reduced neurite outgrowth in PC12 cells. The data are shown as the mean ± SEM values (* indicates *p* < 0.05, meaning a statistically significant difference, while N.S. indicates nonsignificance).

PC12 cells, a cell line from the rat pheochromocytoma of the adrenal medulla, have been extensively used as a mature neural cell model of neurite outgrowth [27,28,29]. In order to further validate the effect of the RhoA signaling pathway on neurite outgrowth, neuronally differentiated PC12 cells were treated with CT04 or Y27632 for 24 h. The results indicated that both CT04 and Y27632 treatments increased neurite outgrowth in neuronally differentiated PC12 cells (Figure 2A–C,G,H). Further studies revealed that the overexpression of constitutively activated RhoA (RhoAQ63L transfection) reduced neurite outgrowth in neuronally differentiated PC12 cells (Figure 2D–F,I,J). Overall, the appearance of neuronally differentiated PC12 cells was consistent with that of DRG neurons. In order to minimize the usage of animals for the primary neuron culture, the neuronally differentiated PC12 cells were used in the following experiments to investigate the potential mechanisms underlying RhoA-mediated neurite outgrowth. Similar to the DRG neurons, the neuronally differentiated PC12 cells in the Blank group were cultured for 3 days longer than those in the Control group to allow expression of the transgene, which resulted in the average length of the neurites in the Blank group becoming longer than those in the Control group.

### 3.3. The RhoA Signaling Pathway Increases Glu-Tubulin Expression in Neuronally Differentiated PC12 Cells

**Figure 3:**
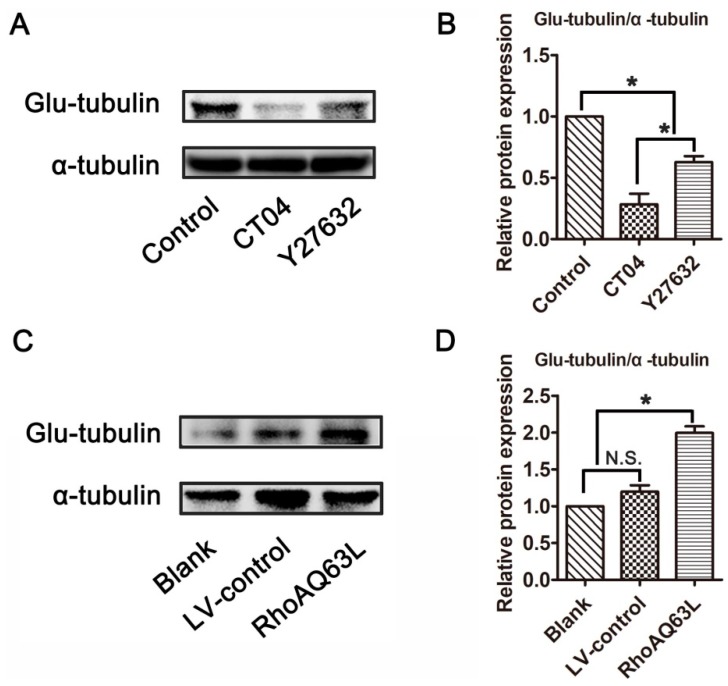
The RhoA signaling pathway elevates the expression of Glu-tubulin in neuronally differentiated PC12 cells. Western blots showed that the level of Glu-tubulin was remarkably downregulated in CT04- or Y27632-treated cells (**A**,**B**). Conversely, the overexpression of constitutively activated RhoA resulted in the upregulation of Glu-tubulin (**C**,**D**). The ratio of Glu-tubulin/total tubulin (α-tubulin) in the control group was normalized to 1. The data are shown as the mean ± SEM values (* indicates *p* < 0.05, meaning a statistically significant difference, while N.S. indicates non-significance).

Microtubule detyrosination is one of the most important factors involved in regulating microtubule dynamics, and detyrosinated tubulin (Glu-tubulin) has been widely utilized as a marker of stabilized microtubules [30,31]. To test whether the RhoA signaling pathway is involved in the regulation of microtubule dynamics, the expression level of Glu-tubulin was detected. Western blotting verified that Glu-tubulin was dramatically reduced in the presence of CT04 or Y27632 (Figure 3A,B). On the contrary, the overexpression of constitutively active RhoA resulted in a significant elevation of Glu-tubulin (Figure 3C,D). Collectively, these results indicate that the RhoA signaling pathway may impact microtubule stability in neuronally differentiated PC12 cells. 

### 3.4. The RhoA Signaling Pathway Inhibits Spastin and p60-Katanin Expression

**Figure 4:**
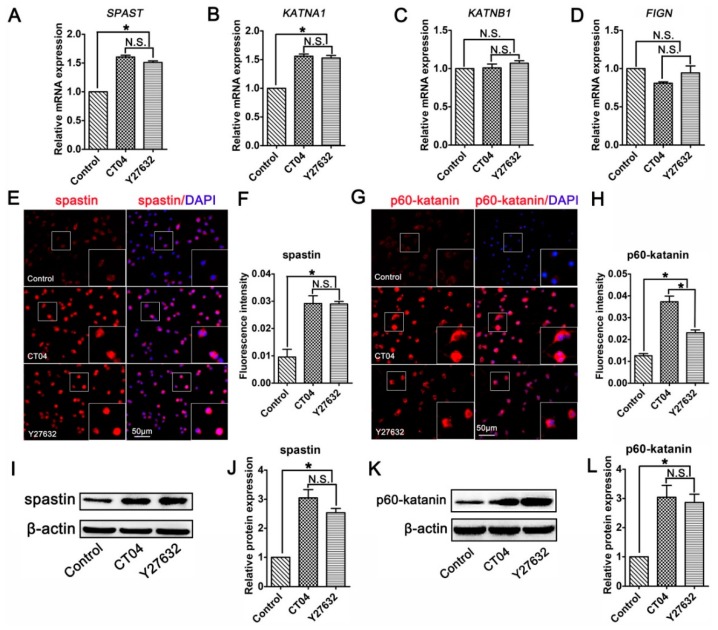
Inhibition of the RhoA signaling pathway upregulates the expression of spastin and p60-katanin in neuronally differentiated PC12 cells. (**A**–**D**) Quantitative RT-PCR analysis demonstrated that the mRNA levels of *SPAST* and *KATNA1* were increased in the presence of CT04 or Y27632, whereas the mRNA levels of *KATNB1* and *FIGN* were not influenced by CT04 or Y27632. (**E**–**H**) Immunocytochemistry and quantification revealed that the immunointensities of spastin and p60-katanin were statistically increased in the CT04- or Y27632-treated cells. (**I**–**L**) A quantitative analysis of Western blots illustrated the different protein levels of spastin and p60-katanin in each group. Both the mRNA and protein levels of the target proteins in the control group were normalized to 1. The data are presented as the mean ± SEM values (* indicates *p* < 0.05, meaning a statistically significant difference, while N.S. indicates non-significance).

Studies have indicated that microtubule dynamics are largely regulated by microtubule-severing proteins [9,32,33]. Moreover, microtubule-severing proteins have been shown to be essential for neurite outgrowth and axon branching [34,35,36,37]. This led us to hypothesize that microtubule-severing proteins might be vital players in the neurite outgrowth directed by the RhoA signaling pathway. To verify this assumption, the mRNA levels of microtubule-severing proteins were investigated. The results of the RT-PCR analysis showed that the addition of CT04 or Y27632 caused elevations in the mRNA levels of both *SPAST* (spastin gene) and *KATNA1* (p60-katanin gene) after 24 h but did not affect the expression levels of *KATNB1* (p80-katanin gene) or *FIGN* (fidgetin gene) (Figure 4A–D). To further validate these findings, immunofluorescence staining and Western blotting were conducted to evaluate the protein levels of spastin and p60-katanin. Immunofluorescence staining suggested that intense spastin and p60-katanin immunoreactivities were stronger in the CT04 and Y27632 groups compared to the control group (Figure 4E–H). Western blotting detection also indicated that the expression levels of spastin and p60-katanin were remarkably upregulated in the presence of CT04 and Y27632 (Figure 4I–L).

**Figure 5:**
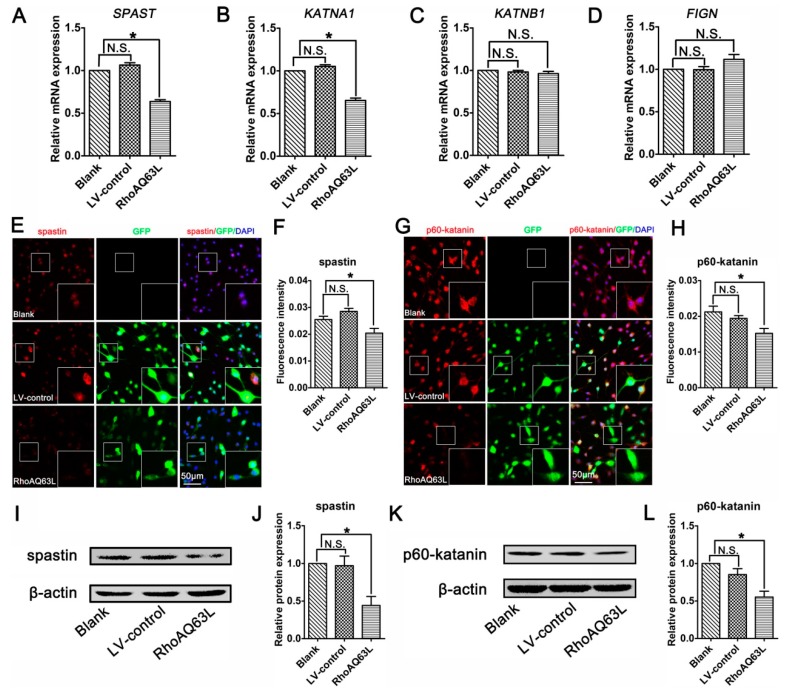
RhoAQ63L transfection decreases the expression of spastin and p60-katanin. (**A**–**D**) Quantitative RT-PCR indicated that the mRNA levels of *SPAST* and *KATNA1* were significantly decreased in the RhoAQ63L group compared with that in the Blank and LV-control groups, while the mRNA levels of *KATNB1* and *FIGN* were not statistically different between groups. (**E**–**H**) Immunocytochemistry revealed that the spastin and p60-katanin expressions were reduced in RhoAQ63L transfected cells. (**I**–**L**) Western blotting illustrated that RhoAQ63L transfection downregulated the expression of spastin and p60-katanin proteins. Both the mRNA and protein levels of the target proteins in the control group were normalized to 1. Data are shown as the mean ± SEM values (* indicates *p* < 0.05, meaning a statistically significant difference, while N.S. indicates nonsignificance).

To confirm the effects of the RhoA signaling pathway on proteins related to microtubule severing, we next analyzed the effects of RhoAQ63L transfection on the expression of microtubule-severing proteins. The RT-PCR analyses revealed that the expression levels of both the *SPAST* and *KATNA1* genes in the RhoAQ63L group were evidently lower than those in the Blank and LV-control groups, whereas the levels of *KATNB1* and *FIGN* were not altered (Figure 5A–D). Moreover, the quantification of immunofluorescence and Western blotting showed that constitutively active RhoA downregulated the expression levels of spastin and p60-katanin (Figure 5E–L). These data led us to hypothesize that spastin and p60-katanin might be involved in the inhibitory effect of the RhoA signaling pathway on neurite outgrowth.

### 3.5. Spastin or p60-Katanin Knockdown Reverses the Positive Effect of CT04 on Neurite Outgrowth

**Figure 6:**
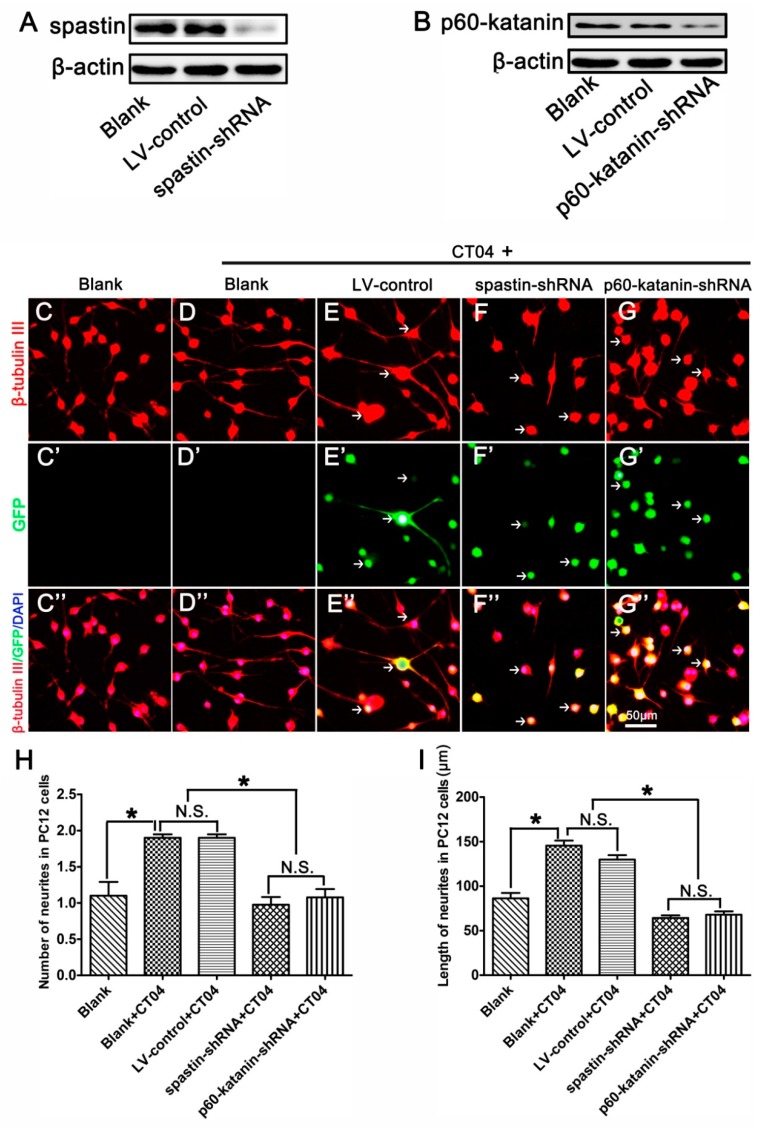
Lentiviral spastin-shRNA and p60-katanin-shRNA blocks the positive effect of CT04 on neurite outgrowth. (**A**) Western blotting demonstrated that spastin-shRNA transfection reduced the expression of spastin compared with the LV-control and Blank groups in differentiated PC12 cells. (**B**) Western blot detection showed that p60-katanin-shRNA transfection decreased the expression of p60-katanin compared with the LV-control and Blank groups. (**C**–**D**, **C′**–**D′**, **C″**–**D″**) The neuronally differentiated PC12 cells were treated with CT04 for 24 h. Representative immunofluorescence staining for β-tubulin III revealed that CT04 treatment induced neurite outgrowth in neuronally differentiated PC12 cells. (**E**–**G**, **E′**–**G′**, **E″**–**G″**) A lentiviral vector targeting spastin or p60-katanin (spastin-shRNA/p60-katanin-shRNA) and a control vector (LV-control) were employed to transfect differentiated PC12 cells. After 5 days, the cells were treated with CT04 for 24 h. Quantitative analyses of the number (**H**) and length (**I**) of the neurites of each cell in different groups. The results revealed that, compared with the LV-control and Blank groups, spastin-shRNA or p60-katanin-shRNA transfection reversed the positive effect of CT04 on neurite outgrowth. The blots were cropped from different parts of the same gel. The data are shown as the mean ± SEM values (* indicates *p* < 0.05, meaning a statistically significant difference, while N.S. indicates nonsignificance).

To further determine whether the roles of RhoA in neurite outgrowth are attributable to spastin and p60-katanin, these two proteins were experimentally knocked down with lentivirus-mediated shRNA before the cells were treated with CT04. A Western blot analysis indicated that the lentivirus-mediated shRNA transfection dramatically knocked down the expression of spastin and p60-katanin compared with the LV-control and Blank groups (Figure 6A,B). Subsequently, β-tubulin III immunostaining was conducted to analyze the neurite outgrowth. As shown in Figure 6C–I, compared to the blank control cells that were not treated with CT04 and lentivirus transfection, the number and length of neurites were significantly increased by CT04 treatment of the non-transfected cells (Blank+CT04 group) and the control virus transfected cells (LV-control+CT04 group). Moreover, the LV-control+CT04 group showed no statistical difference from the Blank+CT04 group, which means that lentivirus transfection did not affect neurite outgrowth. However, the lentivirus-mediated spastin or p60-katanin knockdown (spastin-shRNA+CT04 group or p60-katanin-shRNA+CT04 group) significantly reversed the positive effect of CT04 on neurite outgrowth when compared to the LV-control+CT04 group.

### 3.6. The Regulation of Spastin and p60-Katanin Expression by the RhoA Signaling Pathway is Independent of p38 or AKT

**Figure 7:**
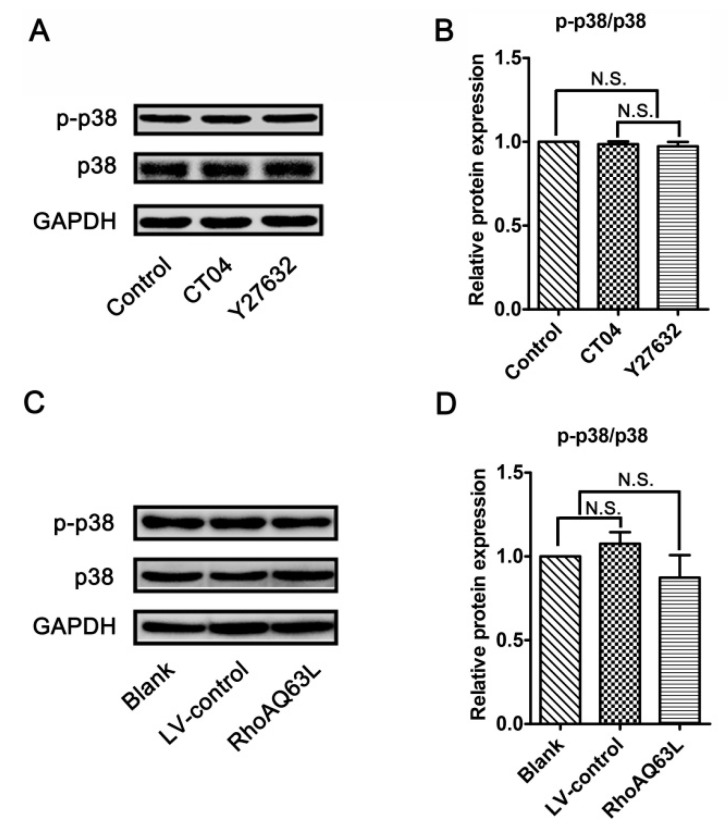
The RhoA signaling pathway does not alter the expression and activation of p38 MAPK in neuronally differentiated PC12 cells. (**A**–**D**) Western blots showed that the levels of total p38 MAPK (p38) and phosphorylated p38 MAPK (p-p38) were not significantly regulated by CT04, Y27632 treatments, or the overexpression of constitutively activated RhoA (RhoAQ63L). The protein level in the control group was normalized to 1. The data are shown as the mean ± SEM values (N.S. indicates nonsignificance).

Since the RhoA/ROCK pathway mediates the morphological changes of the spinal microglia involved in p38 MAPK activation [38], and because neurite outgrowth is also a morphological change, we wondered whether p38 MAPK activation plays role in the regulation of spastin and p60-katanin expression by the RhoA signaling pathway. Therefore, the protein levels of the total p38 MAPK (p38) and phosphorylated p38 MAPK (p-p38, to indicate the activation of p38) were detected by Western blotting. As shown in Figure 7, neither p38 nor p-p38 experienced any significant changes after the neuronally differentiated PC12 cells were treated with CT04, Y27632 (to inactivate RhoA or ROCK) or transfected with LV-RhoAQ63L (to overexpress activated RhoA). These results directly exclude the possibility that p38 participates in RhoA regulation in the downstream molecules. In other words, these data demonstrate that the regulation of spastin and p60-katanin expression by the RhoA signaling pathway is independent of p38.

**Figure 8:**
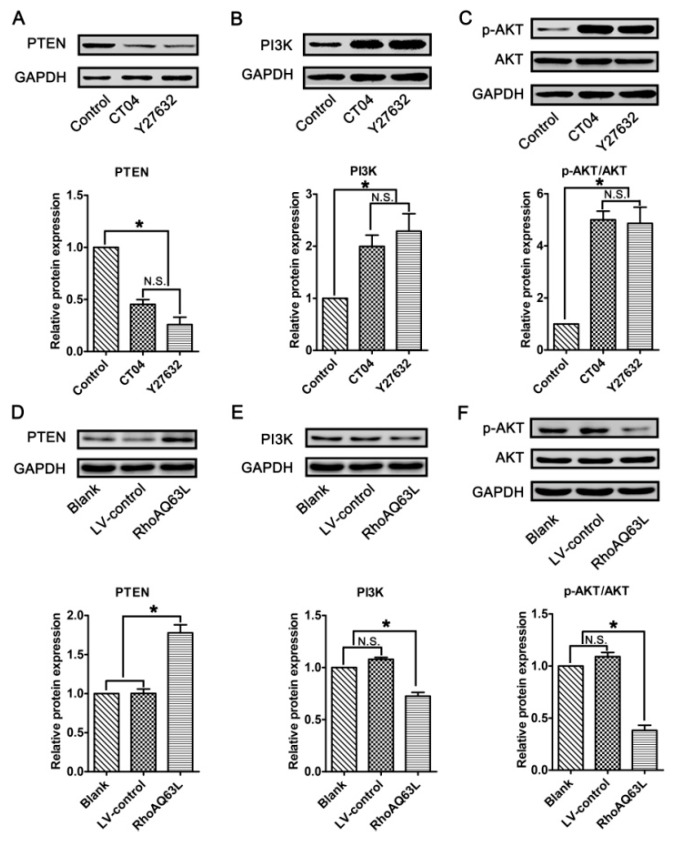
The RhoA signaling pathway negatively regulates the AKT signaling pathway in neuronally differentiated PC12 cells. (**A**–**C**) Western blots showed that the level of PTEN was remarkably downregulated, while PI3K and p-AKT were dramatically upregulated in CT04- or Y27632-treated cells. (**D**–**F**) Conversely, the overexpression of constitutively activated RhoA resulted in the upregulation of PTEN and the downregulation of PI3K and p-AKT. The protein level in the control group was normalized to 1. The data are shown as the mean ± SEM values (* indicates *p* < 0.05, meaning a statistically significant difference, while N.S. indicates non-significance).

**Figure 9:**
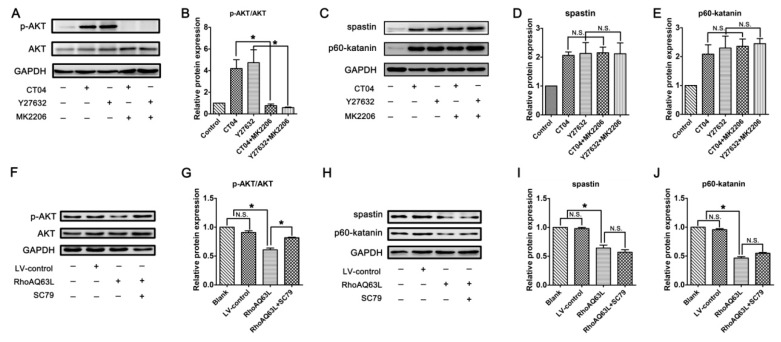
Inactivation or activation of AKT does not reverse the regulation of spastin and p60-katanin by the RhoA signaling pathway. Western blots showed that MK2206 significantly reversed the effects of CT04 and Y27632 treatment on the upregulation of p-AKT (**A**,**B**) but did not reverse the upregulation of spastin and p60-katanin by CT04 and Y27632 (**C**–**E**). The RhoAQ63L-induced suppression of p-AKT was obviously restored by SC79 treatment (**F**,**G**), while the RhoAQ63L-induced suppression of spastin and p60-katanin could not be restored by SC79 (**H**–**J**). The protein level in the control group was normalized to 1. The data are shown as the mean ± SEM values (* indicates *p* < 0.05, meaning a statistically significant difference, while N.S. indicates nonsignificance).

In neurons, PTEN is a main substrate of ROCK. PTEN can inactivate AKT to suppress neuronal survival [39]. Herein, we attempt to assess the possibility that the AKT pathway controls spastin and p60-katanin gene expression during neurite outgrowth. In order to address this issue, PTEN, phosphorylated AKT (Ser473) (p-AKT), AKT, and AKT’s critical upstream regulator, PI3K, were detected by Western blotting. As shown in Figure 8A–C, p-AKT was significantly increased in the CT04- and Y27632-treated cells, while the total AKT was unaffected. Furthermore, the expression of PTEN was downregulated in the presence of CT04 or Y27632, while the expression of PI3K was upregulated. A reversed pattern of p-AKT, PTEN, and PI3K levels could be found when the cells were transfected with LV-RhoAQ63L (Figure 8D–F). These data led us to hypothesize that AKT activation might be involved in the biological effects of RhoA on neuronally differentiated PC12 cells. In order to further detect whether RhoA-regulated AKT activation is involved in controlling spastin and p60-katanin expression, we used MK2206 (a specific inhibitor of AKT activation) [40] and SC79 (a specific activator of AKT) [41] to perform rescue experiments. Our data illustrate that MK2206 can significantly reverse the effects of CT04 and Y27632 treatment on the upregulation of p-AKT (Figure 9A,B) but cannot reverse the upregulation of spastin and p60-katanin by CT04 and Y27632 (Figure 9C–E). On the other hand, the RhoAQ63L induced-suppression of p-AKT was obviously restored by SC79 treatment, while the RhoAQ63L-induced suppression of spastin and p60-katanin could not be ameliorated by SC79 (Figure 9F–J). In short, neither the activation nor inactivation of AKT can affect the expression of spastin and p60-katanin. Therefore, we conclude that AKT activation is not involved in controlling spastin and p60-katanin expression.

## 4. Discussion

As is well known, the best-characterized members of the Rho family are classified into three subgroups, the RhoA (RhoA, -B, and -C), Cdc42 (Cdc42, Tc10, and TcL), and Rac (Rac1, -2, and -3 and RhoG) subfamilies. The RhoA subfamily acts as a molecular switch that regulates neuronal development and regeneration by changing between an active (GTP-bound) and inactive (GDP-bound) form [42,43]. Previous studies have shown that RhoA activation induces growth cone collapse and neurite outgrowth inhibition, which in turn leads to axonal regeneration failure [42,44,45]. Furthermore, a large amount of evidence has demonstrated that RhoA is a convergence point for the intraneuronal signaling pathway related to extracellular inhibitory factors [11,12,13]. Therefore, the RhoA pathway has been widely accepted as an important therapeutic target to enhance axon regeneration after nervous system injury [17,18,19,46]. Thus, the molecular mechanisms of the RhoA-mediated suppression of neurite outgrowth need to be further elucidated to improve their therapeutic effects following nervous system injury.

Both microfilament and microtubule dynamics are prerequisites for neurite outgrowth. However, existing studies have attributed the functions of the RhoA pathway on neurite outgrowth to its regulatory function in actin filament assembly and stabilization [47,48]. Whether RhoA regulates neurite outgrowth through the modulation of microtubule dynamics and its potential mechanisms has not yet been reported, although some evidence has indicated that the RhoA pathway is involved in the regulation of microtubule dynamics in a variety of cells [21,22]. The present study is the first to detect the effects of the RhoA pathway on neurite outgrowth in primary cultured DRG neurons and neuronally differentiated PC12 cells. To inactivate the RhoA pathway, CT04 (a widely used RhoA inhibitor) and Y27632 (a widely used inhibitor of the downstream effector of RhoA, ROCK) were used to treat the subjected cultures. Quantitative analyses demonstrated that the CT04 and Y27632 treatments remarkably increased the number and length of neurites in both the DRG neurons and PC12 cells. To further verify our experimental conclusions, we also transfected the subjected cultures with a RhoAQ63L lentiviral vector. RhoAQ63L is a GTPases-deficient mutant. It cannot hydrolyze GTP, and as a result, the transfected cells can overexpress the constitutively GTP-bound RhoA. Following this, the number and length of the neurites in both the DRG neurons and PC12 cells were dramatically reduced. Thus, based on the data obtained from the negative (inhibition by CT04 and Y27632) and positive (overexpression of the activated RhoA) experiments, we conclude that the RhoA pathway negatively modulated neurite outgrowth, which is consistent with current common views. Additionally, these data lead us to believe that neuronally differentiated PC12 cells could be used in a cell model to assay neurite-outgrowth-related mechanisms, which is another common view. To explore potential molecular mechanisms, abundant cells were needed to isolate the protein and RNA. We used neuronally differentiated PC12 cells to perform further experiments, rather than using DRG neurons, in order to minimize animal usage.

Since detyrosinated tubulin (Glu-tubulin) has widely been utilized as a marker for stabilized microtubules [30,31], the expression level of Glu-tubulin was detected to determine whether the RhoA signaling pathway is involved in the regulation of microtubule dynamics. Our data verified that Glu-tubulin was dramatically reduced in the presence of CT04 or Y27632 but was significantly elevated after the cells were transfected with the RhoAQ63L lentiviral vector. These results indicate that the RhoA signaling pathway could increase microtubule stability. Microtubule dynamics entail a balance of microtubule stabilization and destabilization, regulated by various factors, including microtubule-severing proteins [9,32]. Previous studies have demonstrated that the upregulation of microtubule-severing proteins favors neurite outgrowth [35,49,50], so our further experiments mainly aimed to answer the question of whether RhoA regulates neurite outgrowth via the regulation of the expression of microtubule-severing proteins.

Within the family of microtubule-severing proteins, there are three well-known members: spastin, katanin, and fidgetin [51]. Spastin, encoded by the *SPAST* gene, is responsible for severing the stable domain of microtubules and is able to generate multiple short microtubules from longer microtubules by severing the stable domains of microtubules [52]. Short and free microtubules and the dynamic remodeling of microtubules are thought to be important for growth cone migration and neurite outgrowth [35,53]. Several studies have revealed that spastin plays a key role in the growth of neurites [35,50,54]. Katanin is a heterodimer, consisting of the p60 enzyme subunit (encoded by the *KATNA1* gene) and the p80 enzyme subunit (encoded by the *KATNB1* gene); p60 is involved in the actual severing, whereas the p80 subunit has no severing activity, acting instead to regulate the severing activity of p60-katanin [52]. It has been reported that p60-katanin not only promotes neurite growth but also facilitates the branching of these neurites [36,49]. Fidgetin, encoded by the *FIGN* gene, has a preference for severing the labile domain of microtubules [55]. To date, the relative contributions of fidgetin to neurite growth remain controversial [37,55]. Following the RT-PCR, Western blotting, and immunocytochemistry analyses, the data presented in this study illustrate that inactivating RhoA and its downstream effector ROCK by CT04 or Y27632 could significantly increase the mRNA and protein levels of spastin and p60-katanin. In contrast, following the overexpression of constitutively activated RhoA by RhoAQ63L lentivirus transfection, the mRNA and protein levels of spastin and p60-katanin were dramatically decreased. Meanwhile, the effects of RhoA on the expression of p80-katanin and fidgetin were excluded in the RT-PCR assessment. Based on these findings, we hypothesize that the RhoA pathway could modulate microtubule dynamics by regulating the expression of spastin and p60-katanin, which in turn leads to the inhibition of neurite outgrowth. To further verify this hypothesis, we experimentally and separately knocked down the expression of spastin and p60-katanin in neuronally differentiated PC12 cells, after which the cells were treated with CT04. The results demonstrated that both spastin-shRNA and p60-katanin-shRNA transfection could significantly reverse the positive effect of CT04 on neurite outgrowth. In addition to the above key results, we also found that p38 and AKT, two reported downstream effectors of the RhoA/ROCK pathway in neural tissue, were not involved in controlling spastin and p60-katanin expression during neurite outgrowth. The deeper underlying mechanisms remain to be studied further.

Overall, the present study elucidated that the RhoA signaling pathway modulates neurite outgrowth by regulating the expression levels of spastin and p60-katanin. This finding provides new insight into the molecular mechanisms behind the RhoA-induced negative regulation of neurite outgrowth, which means that the RhoA signaling pathway inhibits neurite outgrowth not only by regulating actin filament dynamics but also through the involvement of spastin- and p60-katanin-modulated microtubule dynamics. Since the inhibition of the RhoA pathway has been widely suggested as a prospective therapeutic strategy for axonal regeneration after nervous system injury, fully understanding the molecular mechanisms of the RhoA-mediated suppression of neurite outgrowth is of critical importance. We believe that the current findings may provide a novel way to explain the roles of RhoA in neuronal development and regeneration.
